# Partial omission of bleomycin for early‐stage Hodgkin lymphoma patients treated with combined modality therapy: Does incomplete ABVD lead to inferior outcomes?

**DOI:** 10.1002/jha2.1

**Published:** 2020-02-07

**Authors:** Jillian R. Gunther, Chelsea C. Pinnix, Gordon R. Glober, Kaitlin M. Christopherson, Penny Fang, Hun Ju Lee, Sairah Ahmed, Raphael E. Steiner, Ranjit Nair, Paolo Strati, Sattva S. Neelapu, Loretta J. Nastoupil, Bouthaina S. Dabaja

**Affiliations:** ^1^ Department of Radiation Oncology The University of Texas MD Anderson Cancer Center Houston Texas; ^2^ University of Central Florida College of Medicine Orlando Florida; ^3^ Department of Lymphoma & Myeloma The University of Texas MD Anderson Cancer Center Houston Texas

**Keywords:** chemotherapy, Hodgkins lymphoma, radiotherapy

## Abstract

Classical Hodgkin lymphoma (HL) patients achieve excellent outcomes; therefore, treatment de‐escalation strategies to spare toxicity have been prioritized. In a large randomized trial of early‐stage HL patients, omission of chemotherapeutic agents including bleomycin from the standard ABVD (doxorubicin, bleomycin, vinblastine, dacarbazine) regimen was not found to be noninferior; however, the effect of partial omission is unknown. We investigated the effect of bleomycin omission on outcome for 150 early‐stage HL patients. At 8 years, freedom from relapse was 99% for both patients who received complete or incomplete bleomycin, which is reassuring for patients requiring bleomycin omission due to toxicity.

## 1 INTRODUCTION

Given the excellent prognosis of classical Hodgkin lymphoma (HL) patients, therapy has been progressively de‐escalated to minimize treatment‐related toxicity. Large HL‐randomized trials have explored bleomycin omission from all cycles [1] or later cycles [2] of the standard ABVD (adriamycin, bleomycin, vinblastine, dacarbazine) regimen, but noninferiority could not be clearly established. For patients planned for combined modality therapy (CMT) with chemotherapy and radiation therapy (RT), oncologists may omit bleomycin from later cycles due to concern for increased risk of pulmonary toxicity. We aimed to explore the effect of full or partial omission of bleomycin on outcomes for early‐stage HL (ESHL) patients treated with CMT.

## 2 MATERIALS AND METHODS

With IRB approval, we reviewed our institutional records of all ESHL patients treated between 2002 and 2013. Patients with initial positron emission tomography‐computed tomography (PET‐CT) imaging who received more than two cycles of ABVD and RT were included. Patients treated with ABVD for all cycles comprised the complete bleomycin (CB) cohort. Those who had bleomycin omitted from at least one cycle of chemotherapy were included in the incomplete bleomycin (IB) group. Patients receiving only part A of a cycle were recorded as .5.

The five‐point scale (FPS) [3] was not routinely used in assessment of PET‐CT imaging. Therefore, patients were considered to have a negative interim PETCT if language such as “complete metabolic remission” or “complete resolution of FDG avidity” was used. All other interim PETCTs were reviewed independently by two radiation oncologists (JRG, CCP) and an FPS score was assigned. FPS scores of 1‐3 were considered negative.

We compared characteristics and treatment‐related variables for the CB and IB administration groups using Fisher's exact test and Wilcoxon rank sum. All statistical analyses were performed using JMP version 14 (SAS Institute, Cary, NC, USA). Overall survival (OS) and freedom from relapse (FFR) were estimated using Kaplan‐Meier analysis.

## 3 RESULTS

The study included 150 patients; 77 (51%) received CB (Table 1). Early favorable (per German Hodgkin Study Group [GHSG] definition [4]) patients comprised 21% of the cohort (n = 32). Clinical factors of age (*P* = .08), GHSG status (*P* = .84), bulky disease (*P* = .86), and extranodal disease (*P* = .28) did not differ between the CB/IB groups.

**Table 1:** Patient, disease, and treatment characteristics

	All pts (n = 150)	CB (n = 77)	IB (n = 73)	*P*
Age				
Median	32	31	34	.08
Range	18‐78	18‐67	18‐78	
Sex				
Male	66 (44%)	38 (49%)	28 (38%)	.19
Female	84 (56%)	39 (51%)	45 (62%)	
Stage				
I	28 (19%)	15 (19%)	13 (18%)	.84
IIA	122 (81%)	62 (81%)	60 (82%)	
Risk factors				
≥3 Sites	85 (57%)	40 (52%)	45 (62%)	.25
Bulky disease	50 (34%)	27 (35%)	24 (33%)	.86
Elevated ESR^a^	16 (of 51) (31%)	10 (of 30) (33%)	6 (of 21)	.77
Extranodal disease	8 (5%)	6 (8%)	2 (3%)	.28
B symptoms	25 (17%)	14 (18%)	11 (15%)	
GHSG risk group				
Early favorable	32 (21%)	17 (22%)	15 (21%)	.84
Early unfavorable	118 (79%)	60 (78%)	58 (79%)	
Chemotherapy				
Cycles of ABVD (median, range)	4 (3‐6)	4 (3‐6)	4 (4‐6)	.03
Cycles of bleomycin (median)	4 (0‐6)	4 (3‐6)	3 (0‐5)	<.001
Radiation				
Median (range)	30.6 Gy (20‐40)	30.6 Gy (20‐39.6)	30.6 Gy (20‐40)	.99
Interim PET				
Positive^b^	7 (5%)	5 (7%)	2 (3%)	<.001
Negative	113 (75%)	48 (62%)	65 (89%)	
Not performed, missing reports/images	30 (20%)	24 (31%)	6 (8%)	
Rationale for bleomycin omission		n/a		
Bleomycin pulmonary toxicity			33 (45%)	
Expected to receive radiation therapy			26 (36%)	
Age/clinical comorbidities			7 (10%)	
Other			7 (10%)	

Abbreviations: ESR, erythrocyte sedimentation rate; GHSG, German Hodgkin Study Group.

a Elevated ESR ≥30 if B symptoms, and ≥50 if no B symptoms.

b Deauville (Five Point Scale) scores of 4, 4×, and 5 were considered positive.

All patients received a median 4c of ABVD with a significantly higher number of total cycles for those in the CB versus IB groups (median 4.0 vs 3.0, respectively, *P* < .001). For the IB group, bleomycin was omitted for: 1c (n = 34), 2‐3c (n = 27), or ≥4c (n = 12). Cited reasons for IB were bleomycin toxicity (n = 33, 45%), expectation to receive RT (n = 26, 36%), age/clinical condition (n = 7, 10%), and unknown/other (n = 7, 10%). Positive interim PETCTs were observed in five CB and two IB patients. A significantly higher proportion of patients in the CB group versus IB group had negative interim PETCT imaging compared to patients with positive or missing PET‐CTs (*P* = <.001). For all patients, the median RT dose was 30.6 Gy, and dose did not differ between groups (*P* = 0.99).

Median follow‐up was 7.4 years for all patients (6.7 years for CB vs 8.5 years for IB, *P* = 0.17). Three patients experienced relapse (two CB and one IB); all were successfully salvaged with chemotherapy and autologous stem cell transplant. One patient recurred within the mediastinal RT field. One patient subsequently developed therapy‐related acute myelogenous leukemia.

**Figure 1:**
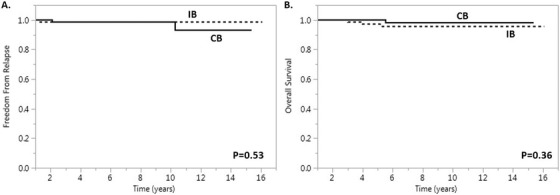
Freedom from relapse (A) and overall survival (B) of ESHL patients treated with incomplete bleomycin (IB) and complete bleomycin (CB)

Of the four deaths (one CB and three IB) reported, none were known to be disease or therapy related, with cause attributed to rectal cancer, glioblastoma, respiratory failure (no bleomycin), and an unknown cause. Eight‐year FFR was 99% for all patients and was not statistically different between the CB and IB cohorts (99% and 99% for CB vs IB patients, *P* = .53). Eight‐year OS was 98% and 96% for CB versus IB patients, respectively (*P* = .36) (Figure 1).

## 4 DISCUSSION

In this study, we investigated the effect of full or partial bleomycin omission from the standard ABVD regimen for ESHL patients receiving CMT. We found that patients who received regimens with CB or IB both achieved excellent outcomes in terms of FFR and OS.

The pulmonary toxicity of bleomycin is well established [5, 6], and this concern has motivated the randomized trials that examined the full or partial omission of the agent from the ABVD backbone. A large randomized trial of ESHL patients examined the effect of omitting dacarbazine, bleomycin, or both from standard ABVD therapy [1]. Five‐year freedom from treatment failure was clearly inferior when dacarbazine was omitted (81.4% vs 93.1%). Noninferiority of AVD compared to ABVD could also not be confirmed (89.2% vs 93.1%). However, the rates of grade III or IV toxicity were lower (26% vs 33%) with only bleomycin omission. Johnson et al published a randomized trial of advanced‐stage HL patients attempting to de‐escalate treatment with the omission of bleomycin for the final four chemotherapy cycles for patients achieving a complete metabolic response after two cycles of initial ABVD therapy. While outcomes were excellent for the de‐escalated group, the 3‐year progression‐free survival was technically not noninferior for the group treated with bleomycin omission [2].

In our study, provider rationales for omitting bleomycin varied, but several common themes emerged. Most often, providers chose to discontinue bleomycin due to symptoms or signs suggestive of pulmonary toxicity which included cough, shortness of breath, radiographic pulmonary changes, and/or worsening pulmonary function tests. Bleomycin administration was also terminated prematurely for many patients who were planned to receive consolidation RT. There may be less concern regarding inferior outcomes with bleomycin omission from initial chemotherapy for patients planned to receive CMT. There is also possible theoretical concern for increased pulmonary toxicity in patients where the radiation treatment field would necessitate partial lung treatment. However, among HL patients that experience bleomycin toxicity, prior studies do not support an increased risk of radiation pneumonitis after mediastinal RT [5, 7].

Finally, age or comorbid conditions often influenced the decision to omit bleomycin from all or part of the ABVD regimen. The median age of the IB group was slightly higher than the CB group, suggesting that providers may be more inclined to omit bleomycin in older patients. Studies have investigated the toxicity of the ABVD regimen in elderly patients, with focus on bleomycin‐induced lung toxicity [8, 9]. One study analyzed patients 60 years or older who were treated on the HD10 and HD13 trials and compared those who received two cycles of ABVD or AVD with those who received four cycles of ABVD, all followed by RT. Grade III‐IV adverse events and bleomycin‐induced lung toxicity were higher in those patients receiving four cycles of therapy. The authors conclude that more than two cycles of bleomycin led to a high risk of severe toxicity for older HL patients. Another study reported outcomes and toxicity for 147 patients aged 60 or above who received ABVD treatment for HL. As 14% of deaths were related to lung toxicity, the authors recommend bleomycin dose reduction or removal [9]. A phase I trial attempted to replace bleomycin with lenalidomide for Hodgkin lymphoma patients aged 60 years or above, and concluded that this approach was feasible and highly effective [10].

Although the toxicity concerns are valid, thus far published randomized data do not support the noninferiority of full bleomycin omission from the ABVD regimen, and partial bleomycin omission for early‐stage patients receiving CMT has not been investigated. Our data suggest that partial bleomycin omission for ESHL patients treated with consolidative RT may not negatively impact outcome. However, our study has several limitations, including the small study population and low event rate, which preclude more robust statistical analyses and subgroup comparisons. It is also limited by the retrospective nature and the provider biases that impacted decisions regarding bleomycin omission. There was a significant difference in the number of total cycles of chemotherapy that patients in the IB and CB groups received, suggesting that providers had concern for increased pulmonary toxicity with the administration of a full six cycles of bleomycin. However, it is also possible that, for patients requiring early bleomycin omission, providers had concern for inferior outcomes and therefore decided to give additional cycles. Moreover, a significantly higher proportion of patients had interim PET‐CTs read as negative in the IB compared to the CB group, suggesting that providers were more willing to omit bleomycin after having the assurance of a negative interim PETCT. It is also possible that patients who received IB had an overall more favorable prognosis (given the interim PETCT negativity) compared to the CB group; however, the large number of patients in the CB group without interim PETCT results complicates this comparison.

## 5 CONCLUSION

ESHL patients treated with CMT achieve excellent outcomes, even with IB as part of ABVD. While CB omission cannot be concluded to be noninferior, these results are reassuring for patients requiring bleomycin omission due to toxicity. Future randomized trials could examine the role of PET‐directed partial bleomycin omission for ESHL patients.

## AUTHOR CONTRIBUTIONS

Jillian R. Gunther performed the research, analysis, and interpretation of the data, and drafted the manuscript. Chelsea C. Pinnix and Bouthaina S. Dabaja performed the research, contributed to manuscript writing, provided critical revisions and interpretation of data, and reviewed/approved the manuscript. Gordon R. Glober and Kaitlin M. Christopherson performed clinical data abstraction and reviewed/approved the manuscript. Penny Fang, Sairah Ahmed, Raphael E. Steiner, Ranjit Nair, and Paolo Strati provided critical revisions and interpretation of data, and reviewed/approved the manuscript. Hun Ju Lee treated patients included in the study and reviewed/approved the manuscript. Sattva S. Neelapu and Loretta J. Nastoupil treated patients included in the study, provided critical revisions and interpretation of data, and reviewed/approved the manuscript.

## CONFLICT OF INTEREST

C.C.P. reports support from Global Oncology One, *The International Journal of Radiation Biology Physics*, and trial support from Merck. She reports no personal funding related to this work. J.R.G. reports funding from RSNA, unrelated to this work. All MD Anderson Cancer Center co‐authors were supported in part by the NIH Grant. Otherwise, the authors have no conflict of interest.
